# Answers in search of questions: what does the comparison of COVID19 data among regions in Northern Italy tell us?

**DOI:** 10.1017/S1744133120000377

**Published:** 2020-09-04

**Authors:** Luke B. Connelly, Stephen Birch

**Affiliations:** 1 Centre for the Business and Economics of Health, University of Queensland, Brisbane, Australia; 2 Faculty of Economics and Statistics, University of Bologna, Bologna, Italy; 3 Centre for Health Economics, University of Manchester, Manchester, UK

## Abstract

Since the outbreak of the COVID-19 pandemic, discussions about the capabilities of health and social systems to control and contain infectious diseases have been reignited. In *Resilient Managed Competition During Pandemics: Lessons from the Italian Experience*, Costa-Font, Turatti and Levaggi ask whether or not institutional differences between the managed competition (MC) systems in three of Italy's regions may have affected their performance – and hence, population health outcomes – during the pandemic. Fuchs (2000) previously argued that institutional arrangements not only ‘*matter*’, but also sometimes ‘matter a great deal’ (p. 149, emphasis in original) and this may be particularly true in emergencies.

Since the outbreak of the COVID-19 pandemic, discussions about the capabilities of health and social systems to control and contain infectious diseases have been reignited. In *Resilient Managed Competition During Pandemics: Lessons from the Italian Experience*, Costa-Font, Turatti and Levaggi ask whether or not institutional differences between the managed competition (MC) systems in three of Italy's regions may have affected their performance – and hence, population health outcomes – during the pandemic. Fuchs (2000) previously argued that institutional arrangements not only ‘*matter*’, but also sometimes ‘matter a great deal’ (p. 149, emphasis in original) and this may be particularly true in emergencies.

The authors focus on differences in the MC models adopted by three neighbouring regions of northern Italy: Emilia-Romagna, Veneto and Lombardy. Emilia-Romagna and Veneto adopted centralized models in which funding and provision are strongly integrated. Lombardy, by contrast, has a system of ‘decentralized managed competition’ in which public and private providers compete against each other to supply the health services to be offered, and the purchaser (insurer) function is separate. The authors' main thesis is that decentralization (as in Lombardy) leads to worse performance in a pandemic than more centralized MC models, advancing several conceptual arguments to support the hypothesis and presenting comparative data on hospital ownership, COVID-19 infection-fatality rates and swabs per capita, for the three regions.

The authors argue that unless ‘integration is in place, MC encompasses limited incentives for providers to cooperate’ (p. 2), adding that ‘organizational models based on competition without an integrated authority do not face the incentives for a swift response’ which can cause ‘a delayed reaction’ (p. 4) and result in more fatalities. They also argue that high transactions costs may accompany contracting in decentralized systems, noting that the regional health authority (RHA) in Lombardy had to engage in ‘tedious negotiations’ of contracts with private hospitals to provide for the treatment of COVID-19 patients. These questions pertain to the ‘make-or-buy’ decision for Italian RHAs, the problems of codification and complete contracting on markets if the ‘buy’ decision is made, transactions costs, the ownership of strategic assets and the susceptibility of the payer to the ‘hold-up’ problem (see, e.g. Besanko *et al*., 2016). All are important considerations and deserve analysis in the context of pandemic preparedness of health and social care systems.

Lombardy's situation was unique, both within Italy and worldwide. COVID-19 hit Lombardy first and much earlier than in the neighbouring regions and it appears to have been circulated – undetected or undiagnosed – in the region since January 2020. Doctors in the region treating pneumonia due to CoV-2-SARS did not recognize the cause and, at the time, the virus had erroneously been thought to be contained to China (Winfield, 2020). Consequently high levels of undetected community transmission are expected to have occurred prior to the identification of the first case on 20 February (Odone *et al*., 2020). Subsequently, high infection rates overwhelmed hospitals in the region, contributing to the high case-fatality rate that Costa-Font *et al*. report in Table 1 of their article. Contact tracing started on 21 February; although ‘patient zero’ was not identified, a cluster in Codogno was, and within 1 week there were 530 confirmed cases in the region (Cereda *et al*., 2020).

The growth of cases in Lombardy was immediate, rapid and exponential: by 8 March, 5830 cases had been detected (Cereda *et al*., 2020). Odone *et al*. (2020) argue that these factors, along with a ‘possibly delayed public health response’ (p. e310), render the case-fatality rates of little epidemiological value. We are inclined to agree, mainly for the reasons Odone *et al*. (2020) outline, but also because of differences in the rates of testing between the regions (leading to different reported numbers of cases and case rates) and the possibility of confirmation bias during a pandemic leading doctors to over-code deaths as due to COVID-19, that may have been due to other causes with similar presentations. Had another (more integrated) region experienced the ‘initial conditions’ that Lombardy experienced, would the results have been much different? It is difficult to isolate integration as a driver of the deaths data presented in Table 1.

Interestingly, and with the *caveats* they make clear, Odone *et al*. (2020) conducted an exercise in which they computed the CFRs for a 30-day period, from the onset of the first confirmed case, for a number of regions internationally and compared them with Lombardy's CFR. Their comparison shows that the CFRs for New York, USA and Madrid Comunidad, Spain were 81.2 and 77.1 per 100,000 cases, respectively, compared to Lombardy's CFR of 41.4, *notwithstanding* its first-mover, informational, disadvantage. The rates they computed for two other outbreaks in Europe in Île-de-France, France and the Greater City of London, UK, were 26.9 and 23.0, respectively.

Finally, we note that there has been widespread reporting of mishandling of the crisis both in integrated and non-integrated payer-provider health systems. In the UK, there has been heavy criticism of the handling of the virus including, but certainly not limited to, the devastating outbreaks in aged care facilities (The Guardian, 2020). In Australia, which has a high degree of integration of funding and provision, but also a fairly large private sector, similar criticisms have been levelled at the public health response, especially with respect to outbreaks connected with arriving cruise-ships (see, e.g. Baxendale, 2020), hotel quarantine for returning international travellers (see, e.g. Bashan, 2020) and aged care facilities (see, e.g. Moore, 2020).

We agree that structural issues in the health sector may well have been critical to aspects of the (mis)management of the COVID-19 pandemic; however, it seems ‘heroic’ to measure system performance and attribute performance differences by region exclusively to structural, or principal-agent problems in the health sector, based on non-controlled comparisons of epidemiological data between regions.
